# Cellular Microbiology, 2nd ed.

**DOI:** 10.3201/eid1108.050592

**Published:** 2005-08

**Authors:** Frederick Quinn

**Affiliations:** * University of Georgia, Athens, Georgia, USA

**Keywords:** Cellular microbiology, Pascale Cossart, Patrice Boquet, Staffan Normark, Rino Rappuoli

The field of cellular microbiology is relatively new and incorporates aspects of microbiology and host cellular biology. The first edition of this text, published in 2000, was novel and well received. In general, this new edition is also well written and includes many of the most important recent advances in the field (e.g., microarrays and genome sequencing). The text deals almost exclusively with host cell responses elicited by interactions with pathogens. The editors are top researchers in the field of bacterial cellular microbiology, and they have brought together many new investigators to write chapters in their areas of expertise.

The book's first 2 chapters contain topical background information. These chapters thoroughly cover many of the basic concepts in molecular cell biology and introduce all of the various pathogens (bacterial, viral, and eukaryotic) currently being examined in the popular literature. The organization of the subsequent chapters typically alternates between topics in cell biology and bacterial pathogenesis. For example, chapter 11 describes assembly of the cellular cytoskeleton, while chapter 12 describes the mechanisms used by pathogenic bacteria to manipulate the cytoskeleton. Subsequent chapters provide good coverage of bacterial secretion systems, toxins, and their interactions with the host immune system. Generally, the figures, diagrams, and drawings are well chosen, and the tables contain sufficient detail to demonstrate critical points. This is particularly true for chapters 4–6, which describe the host cell surface properties, how pathogenic bacteria adhere to and enter the host cell, and ultimately how the pathogen induces various types of cell signaling. The final chapters focus on new methods of identifying virulence genes and the use of nonvertebrate hosts, such as plants and insects, to model mammalian infections.

This book has only a few drawbacks. For example, the first 2 introductory chapters are too detailed. In subsequent chapters, the emphasis is placed on bacterial pathogens; only 1 chapter is dedicated to viruses and none to eukaryotic pathogens (only the introductory paragraphs in chapter 1) or to nonpathogenic microorganisms of any kind.

This volume will be an important addition to the resources available to students and researchers in general cell biology or microbiology. Perhaps Internet interactive companion programs and accompanying CDs would be useful with future editions. Because the field is moving so quickly, the authors might consider more frequent updates.

